# Challenges of home visit practice and perceived solutions for improvement in Ghanaian context: perspectives of healthcare professional and community informants

**DOI:** 10.1186/s12961-026-01461-w

**Published:** 2026-09-10

**Authors:** Lillian Akorfa Ohene, Samuel Adjorlolo, Margaretta Gloria Chandi, Cecilia Aryeetey, Adelaide Maria Ansah- Ofei, Moses Aikins, Lydia Aziato

**Affiliations:** 1 Department of Public Health Nursing, University of Ghana, Accra, Ghana; 2 Department of Mental Health Nursing, School of Nursing and Midwifery, University of Ghana, Legon, P. O Box LG 43, Accra, Ghana; 3 Research and Grant Institute of Ghana, Accra, Ghana; 4 GA West Municipal Health Directorate, Accra, Ghana; 5 Department of Health Policy, University of Ghana, Accra, Ghana; 6 Department of Research, Education and Administration, University of Ghana, Accra, Ghana; 7 University of Health and Allied Sciences, Ho, Ghana

**Keywords:** Home visit, Guidelines, Health systems, Ghana, Africa

## Abstract

**Background:**

Home visits conducted by nurses and other health professionals are pivotal in ensuring access to healthcare and social services, particularly for the poor and marginalized in Ghana. This paper presents findings from a study conducted in the first and last quarter’s 2020 among health professionals and key community informants on health service delivery in a rural–urban setting in Accra, Ghana. The study’s specific objectives were to (1) explore the challenges besetting home visit practices in Ghana and (2) solicit participants’ perspectives on measures to improve current home visit practices.

**Methods:**

A qualitative exploratory study was adopted. The study population included community healthcare workers (public health nurses, nurses and community health officers), local community leaders and faith healers group leaders. Audio-recorded data were collected during focus group discussions and stakeholder engagements. Three qualitative analysts from the research team conducted thematic and content data analysis.

**Results:**

Barriers to home visits were identified. Thematic areas under the barriers included “*Physical Challenges, Psychological Challenges and Sociocultural* practices as challenges to home visiting.” The second significant finding was centred on multiple measures to improve home visits from health workers. The themes included “*Resources and Incentives, Safety and Security, and In-service Training.*” These significant findings were validated in the stakeholder meetings.

**Conclusions:**

Multi-factorial challenges impede the practices and reception of home visits in Ghana. Based on the findings, we recommend dedicated home visiting guidelines incorporating cultural nuances and providing salient information on service set-up, logistics, and human and financial resources to streamline home visiting practice.

## Background

Home visits are one of the notable initiatives and efforts designed and implemented to promote access to healthcare delivery [1]. A home visit is a healthcare delivery mode in which trained health professionals visit clients in their homes or environments to render healthcare and psychosocial support services [2]. The Universal Healthcare Coverage (UHC) initiative and Goal 3 of the United Nations Sustainable Development Goals, which focuses on health and well-being, strengthen home visits. Under the UHC concept, countries are admonished to promote access to healthcare services to their citizens, regardless of their demographics and other social determinants of access to healthcare [3].

Home visits are essential in Ghana, and other resource-constrained countries where healthcare delivery is negatively affected by a confluence of human resources, logistics, infrastructure and geopolitical and sociocultural factors and cross-cultural studies have provided evidence suggesting that home visits are a noticeable pillar in a decentralized healthcare system, ensuring that individuals, families, households and communities receive timely, appropriate and quality healthcare underpinned by human rights practices [1, 2, 4]. Home visits are excellent for reaching disadvantaged, vulnerable and marginalized individuals, families, households and communities [5, 6], bridging the equity gap, and enhancing responsiveness to healthcare services [7–9]. Home visits have significantly reduced national disease burden and healthcare costs in conjunction with education, awareness creation, community sensitization, health promotion, disease prevention, follow-ups and contact or case tracing [2].

Home visits are an integral part of Ghana’s primary healthcare delivery strategy, dating back to 1928 when the Public Health Nursing Service was established by the Ministry of Health, Ghana [10]. Home visits were initially reserved for public health and community health nurses; however, efforts to promote universal health amidst increasing population growth and exposure to complicated healthcare problems have incentivized the inclusion of other cadres of the health workforce, such as mental health nurses. Home visits are notably underpinned by the Disease Prevention Life Course Approach Model (DPLCAM), which is run in the Community-based Health Planning Strategy (CHPS) is a healthcare delivery model in Ghana. Community health nurses are re-oriented as Community Health Officers (CHOs) to collaborate with the community health management team to implement community-specific health interventions. Despite its widespread use over the years, DPLCAM contains no clear-cut guidelines on specific home visit activities, making it difficult to evaluate.

Anecdotally, given the broad focus of the DPLCAM, tailoring it to meet the unique needs of home visits is notably challenging. More specifically, the DPLCAM does not prescribe how healthcare practitioners undertaking home visits should provide support and care to clients with diverse health needs. It does not contain specific and relevant information on how home visits should be organized, delivered, monitored and evaluated. Anecdotal reports indicate that home visit staff become helpless while undertaking their home visit activities because they are not sure of the referral pathways in a system where inappropriate referrals can further contribute to a delay in access to healthcare [2]. The lack of home visit-specific guidelines also has the propensity to undermine the collective responsibility and not ignite the political support necessary to create a robust early intervention culture to sustain public investment in this area.

To address the challenges mentioned above and improve and streamline home visit programs in Ghana, a dedicated, stand-alone set of guidelines that prescribe the “dos” and “don’ts” specific to home visits is extremely important. This is also timely amidst increasing concerns over future pandemics or epidemics that will invoke the heavy involvement of home visits and community engagement activities. Indeed, home visit guidelines have been developed to support the organization and delivery of home visit services in other settings [4, 11, 12]. However, the gross functional differences in the organization and delivery of healthcare services across jurisdictions provide impetus to adapt the existing home visit guidelines to increase their responsiveness and sensitivity to the Ghanaian healthcare, geopolitical and sociocultural systems. Home visit guidelines that detail local resources or referral systems available to individuals, families, households and communities will help to improve home visit staff engagement with their clients. Adapting home visit guidelines is the first step to ensuring a quality standard of home visits, providing the empirical basis for future implementation and evaluation of implementation success and challenges.

Although home visitation has been practiced over decades, there is limited empirical insight into the challenges besetting the practice in Ghana. The Alliance for Health Policy and Systems Research, through the Research to Enhance the Adaptation and Implementation of Health Systems Guidelines (RAISE) project, has provided funding to support the adaptation of home visit guidelines in Ghana. The adaptation process involves the exploration and review of existing home visit guidelines, mapping out stakeholders to contribute to the adaptation of home visit guidelines and examining the application of the home visit guidelines in selected communities in Ghana. In this paper, we presented the findings involving the exploration of the existing home visit practices prior to the identification of a practice guideline. We explored issues relating to the existing home visit practices that will benefit home visit guidelines. The specific objectives of the study were (1) to explore the challenges besetting home visit practice in Ghana and (2) to solicit from participants measures to improve current home visit practices.

## Method/design

A qualitative exploratory approach was adopted as part of a broader project to explore the challenges and context solutions in developing a home visit guideline in Ghana. This paper focuses only on the data that explored the barriers to conducting home visit services in five Focus Group Discussions (FGDs) among professionals, community herbal practitioners and key informants from the community. In addition, we conducted three stakeholder engagement meetings with health professionals and administrators, community volunteers, key informants and faith and herbal medicine practitioners. These meetings triangulated the data source and validated the findings from the focus group discussions.

### Study setting

The study was conducted in the Greater Accra Metropolis, specifically in the Ablekuma South District. This setting was purposively selected for this study for two reasons. One, Ablekuma South District communities are mainly low-income settlements characterized by problems of poor sanitation, malnutrition and high teenage pregnancy. It is also noted that the district is currently battling infant health issues and needs community health workers’ urgent attention and interventions. Secondly, as mentioned earlier, this study forms part of a bigger project in which home visit guideline was developed and piloted in the district.

### Study population

The study population for the FGDs comprised Community Health Nurses (CHNs) who conducted home visits and Community Health Officers (CHOs) who were responsible for assisting with home visits in the community (Table 1). Other healthcare personnel, Health Programme Managers (HPM) and other agencies such as the District Assembly Health Partners, the Ministry of Education and community members, mainly herbal and faith healers, formed part of the stakeholder engagement groups (Table 2). Based on the participants’ backgrounds, sample groups were formed, each consisting of a specific group type. The targeted individuals were purposely invited to participate through letters. Some agencies nominated officers with knowledge of the topic, and others volunteered to participate. The participants groups had enough gender mix of male and female representations.

**Table 1:** Demographic data of focus group discussion participants (*N* = 36)

Demographic factors	Number of participants (*N*)
Religion	
Christian	36
Gender	
Male	9
Female	27
Ethnicity	
Akan	17
Ewe	8
Ga	11
Professions	
Traders	7
Artisans	7
Herbal/faith healers	6
Nurses	16
Number of working years	
> 5 years	4
5–10 years	21
11–15 years	3
16 + years	8
Highest education level	
No formal education	2
Basic school	4
High school	7
College	16
Bachelors	7

**Table 2:** Number and affiliations of stakeholders engaged (*N* = 46)

Number of reps	Institutions	Positions
12	Government/public hospitals	Nursing managers and officers
5	Non-profit private health organizations	Directors and chairpersons
12	Government health agencies- Policy, Planning, Monitoring and Evaluation (PPME)	Quality managers, drectors, and chairpersons
6	Community leaders	Opinion leaders, herbal and faith healers
5	Security agencies of health	Public health serviceS officers
6	Nursing schools and colleges	Health educators

### Data collection

Data were collected through five face-to-face focus group discussions and three key informant engagement meetings (Stakeholder engagements). In both sessions, a semi-structured interview guide was used to explore participants’ views on the existing model and the challenges of home visits in their local communities. The research team developed the interview guide on the basis of the outcome of a literature search and environmental scan on home visiting practices and guidelines in the sub-Saharan African sociocultural context. The discussion sessions were held at offices in an office complex of easy accessibility to the participants. All eight authors played diverse roles during the data collection discussion meetings. Participants provided consent to audio record the sessions, which were later transcribed. In a relaxed and carefully moderated atmosphere, participants took turns to share their experiences, views and recommendations. The focus group and key informant meeting sessions lasted 90–120 min. The topic was carefully explored, and all participants had enough opportunity to express their thoughts extensively.

### Data analysis

The data was subjected to a thematic and content data analysis. Three qualitative research scientists in the research team conducted the data analysis. All three analysts analysed the data independently using the Braun and Clarke approach [13]. The analysts studied the transcript from the focus groups and stakeholder discussions to familiarize themselves with the data. The triangulation of the two data sources for analysis was possible as we mostly explored the same perspectives in the different groups. The analysts proceeded to conduct a line-by-line coding to identify the initial codes. The codes were reviewed, grouped, and labelled on the basis of meanings and similarities to form themes. The themes identified followed several groupings and regroupings of the critically compelling emerging codes. The analysts compared independent coders’ findings to evaluate the initial codebook’s reliability. After a series of research meetings, the research team reached a consensus on the final identified themes and subthemes.

### Ethical issues and trustworthiness of the study

The study received ethical approval from the Ghana Health Service Ethical Review Committee (GHS-ERC002/01/2020) and the WHO Ethical Review Committee (ERC.0003303). All participants were allowed to read about the study and to ask any questions or concerns regarding their participation. This ensured that all the participants made informed decisions and signed a consent form to participate in the study. Quality and credibility were assured by training all the Research Assistants (RAs) supporting the study. Multiple functional audio recorders ensured quality data capturing, transcription, and analysis. The project coordinator monitored all meetings to provide accurate and consistent procedures.

## Results

### Demographic characteristics of participants

A total of 82 people participated in this exploratory phase of the study. A seven (7) to 11-member team constituted the focus groups. A total of 36 participants (See Table 1) participated in five focus group discussions. Altogether, 46 participants (See Table 2) participated in three different key informant and stakeholder engagement meetings.

### Themes and subthemes

**Fig. 1:**
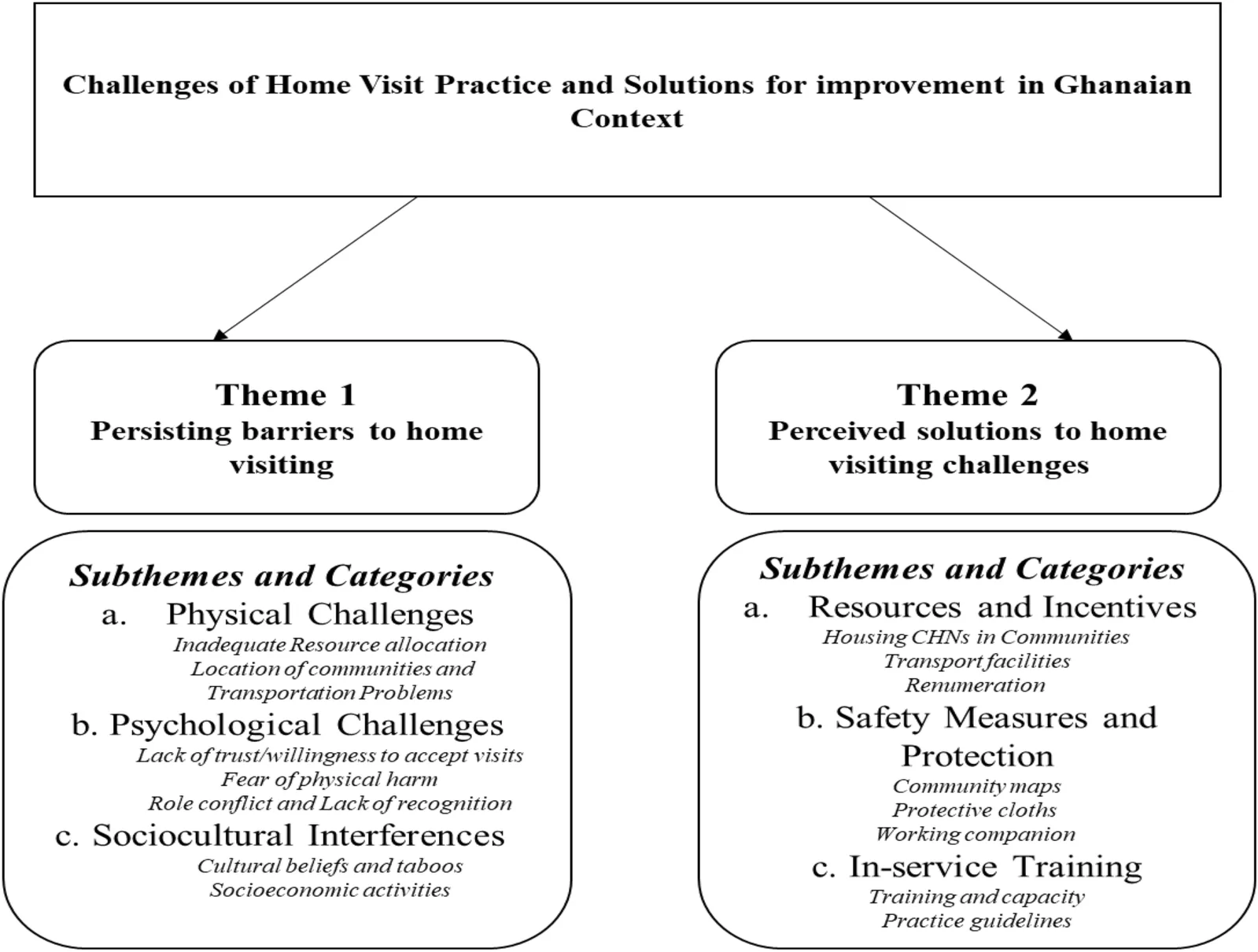
Thematic findings

The findings identified two themes as illustrated in Fig. 1: Persisting Barriers to Home Visiting, with subthemes as *Physical Challenges, Psychological Challenges and Sociocultural practices as challenges to home visits*. The second significant finding was centred on Perceived Solutions to Home Visiting Challenges. The subthemes included, *resources and incentives, safety and security and in-service training.*

### Persisting barriers to home visiting

This central thematic area relates to the challenges affecting the practice of home visits in Ghana. Home visit practices face several challenges such as any other healthcare delivery channel in Ghana. The three subthemes including *physical challenges, psychological challenges, and sociocultural interferences* were identified.

### Physical challenges

This subtheme concerns the challenges home visiting staff encounter regarding resources and logistics, location of the communities, road networks and infrastructure. Community Health Nurses (CHNs) were primarily identified in communities using essential work tools such as visiting bags and vaccine carriers. The CHNs may conduct home visits or other community health visits, such as mobile clinics and health outreach for screening. Children and other priority groups were the populations of interest in such contexts. However, in this study, CHN participants reported needing more basic logistics, including the home visit bags and its contents. It was widely reported that most of the health facilities within the context of this study required essential tools to render basic nursing procedures during a home visit. The commonly mentioned equipment needed was a blood pressure apparatus, thermometers, weighing scales and cotton wool. First aid kits with essential medications for minor ailment treatments were equally lacking. It was also noted that some facilities may need more crucial tools in the home visit bag owing to theft or broken instruments. According to the participants, the CHNs’ protective clothes were also compromised, as the health facilities often had no provision for raincoats, umbrellas or footwear.

In addition, the lack of safe space within the communities to undertake home visit activities further compounded primary health care provision in the districts. The lack of safe, secure working areas for mobile community clinics was another challenge widely reported. Responding to the question “Who must provide the basic resources and logistics for home visiting?” The CHNs and other community health workers mentioned the governments through the district health directorate. They also recalled irregular support from philanthropists, and sometimes, in critical situations, the visiting staff used their income (out of pocket) if they could afford to fund essential care services for their clients. One participant expressed herself as:

“As of now, we do not have any resources. We need something like a thermometer, BP apparatus so that when we go to the field and a client is complaining of a child having temperature, we have to take the temperature of the child, and then if it needs to be sponged, you do it before referring the child to the hospital. But as we speak, we do not have such essential equipment; even our weighing scale is limited. How do we give a family planning agent without checking the client’s Blood pressure? We have the visiting bag, but it is empty. (G1N4).”

Within the context of this project, home visiting assignments were done using a map for CHNs to cover per day and regularly for months or years. Typically, it was a general observation that the nurses were assigned more expansive geographical areas to cover for home visit sessions. According to the participating community members, some areas may be far apart, requiring transportation. Meanwhile, the landscape of some of the communities also posed a challenge to the nurses’ safety. It was reported that some communities had unfriendly terrains; for example, some places were hilly, rocky, densely populated and far apart. Primarily, the visiting team accessed homes by walking within the communities, and as reported earlier, they walked in communities without safety protective gear.

In response to how CHNs navigate the sizeable geographical space to access homes during visits, the CHNs mentioned that they used motorbikes as one of the cheapest and most common means of transport for health visits in rural areas. Nevertheless, the participants quickly said that in the cities with heavy vehicular traffic and its associated higher risks, motorbikes were kept from CHNs, who were primarily women. Others observed that motorbikes were often assigned to males for non-health visit-related duties. Thus, CHNs and, more broadly, women seldom had access to motorbikes for home visits in city settings. Instead, they were paid transportation costs for safer work-related travels. However, the distribution of such incentives and support for home visit activities varied considerably across facilities and jurisdictions and needed to be better documented. These assertions were evident in the data as follows:

“I worked in six other communities before my transfer to Accra…; first, they gave us a motorbike. They trained us on the uses of the motorbike and gave us stipends for fuel. But in Greater Accra, it’s not like that G2N5.”

“The other regions are better. I’ve worked outside Greater Accra before. They provide vehicles for outreach. At this workplace, nothing at all is given for transport purposes (G2N3).”

### Psychological challenges

Psychological challenges were the second subtheme identified, and it is concerned with the lack of trust and or willingness of community members to accept health visitors. The key concerns relate to “fear of physical harm” and “role conflict and lack of recognition.”

Unwillingness on the part of clients to accept visits came up strongly as one of the psychological challenges to the CHNs. Participants reported a sense of mistrust from people about health visitors. For example, in special home visits, CHNs pick contact details from the hospital to follow up on clients with chronic conditions or other exceptional cases. A few CHNs gave accounts that clients often gave incorrect addresses and contact details. It was alleged that some clients refused to pick up calls from the visiting staff on appointment days.

In more complicated cases, some clients may give the contact of family members who may not be accessible. Some clients did not own personal mobile phones. Those who answered calls sometimes provided directions to locations that never existed. For example, a participant said, “Just over there, turn left, brown gate,” such directions were frustrating for health workers. CHN reports demonstrate that not all community members were receptive to nurses visiting their homes, and this lack of ease could be attributed to personal or cultural factors. For example, as one participant noted….

“Mostly, some families do not entertain us in their homes. They never wanted to see us, especially when there is a newborn in the house, and the mother-in-law or the baby’s grandparents are around, and there are some unhealthy practices they wish to do for the newborn, which you [the health worker] will object to. (G1N2).”

Evidence from this study showed that CHNs and other community health workers were at risk of physical and sexual harm. For example, the CHNs reported verbal and physical attacks from relations of clients. Stray dogs attacked nurses under the watch of their owners. A few participants also admitted that some CHNs were sexually assaulted by men in the local communities. These observations followed the sentiment that most CHNs were unarmed young females who could not fight sexual harassment from men in the community. These findings were supported by the statements below:

“A man who prevented us from visiting his wife even came to our office to warn us that he did not want to see us in his area. If he sees us there, he will organize boys to beat us. So, we eventually stopped visiting, and the woman died. (G1N2).”

“Okay. Because of dogs, you have to knock first, especially if it’s a gated house. Recently, we went for home visits just last week together with my colleague. We got to a gated house, and then, as we knocked on the door, a stray dog attacked us. (G2N4).”

The CHNs in this study reported role conflicts with their colleague midwives, especially with the services provided to pregnant women at home. The overlapping role of CHNs in delivering care to priority groups, including pregnant women, may account for these conflicts. Typically, the antenatal booklet serves as a record document for the midwife. However, the CHN, after giving care to pregnant women at home, may be compelled to record findings in the midwives’ record space in the booklets; these were often not welcomed by their midwives. The CHNs may also administer such medications as malaria prophylaxis and Sulphadiazine Pyrimethamine (SP) to pregnant women and need to record this information.

“Even when we wrote in the ANC book, it got to the point that the midwives were unhappy. They actually will query us. So, we had to stop writing in the record books for midwives (G1N5).”

### Sociocultural interferences

The third subtheme, “sociocultural interferences,” describes cultural beliefs, taboos and socioeconomic activities and how these interfere with home visit activities. The views of local people regarding the causes and treatment of diseases interfered with the work of home visit staff. Typically, most community members attach spiritual connotations to health matters and prefer alternative therapy before considering any medical treatment. For instance, participants reported that herbalists only referred to hospitals after they unsuccessfully attempted indigenous non-evidence-based treatments. In addition, most communities have festive and sacred days for other traditional activities. Typically, community members do not welcome health staff on duty on such days.

Furthermore, some religious beliefs discourage modern family planning methods and research evidence. In this regard, several women default on Antenatal Care services (ANC) and continue to give birth at home without the supervision of a skilled midwife. Most of these clients find every reason to avoid the health visitor at home. Instead, they will continue to adhere to old cultural practices which have health implications for community newborns. Some of these practices include the application of hot stone, saliva, ground chalk, gauze and towel on the umbilical cord, as well as keeping the newborn away from the public until an out-dooring ceremony is performed, thereby restricting access to the child for healthcare services.

The social and economic activities in the communities also interfered with home visit activities. For example, in most farming, fishing or commercial communities, accessibility to members for home visit purposes is complicated, as demonstrated in the following participant quote,

“Aside from Tuesdays being their fishing days, some of them, too, have their market days. So, when planning our itinerary, we consider that and do not organize CWC on a Tuesday (G2N1).”

### Perceived solutions to home visiting challenges

The second significant thematic finding relates to overcoming the barriers to home visits to improve primary care services for people in the local communities. Three subthemes emerged under this theme, and these include allocation of *resources and incentives, protection and companionship, and in-service training.*

### Resources and incentives

The participating community members in this study expressed the need for nurses to continue with home visit services. This followed the recognition and appreciation of providing primary health services to the doorsteps of children and older people in communities. Many suggested expansions in benefits and coverage. The concept of Community Health Planning Services (CHPS), where nurses and midwives reside in the community for 24-h services, was re-emphasized. CHNs were open to this and reiterated the readiness of health professionals to conduct practical home visiting services if resources were made available to them. In a summary comment, a CHN said,

“There should be provisions for the listed number of things we need for home visits. Whether government or non-government organizations or the community to aid us in rendering our services. (G1N2)”

Further findings revealed interest in more male nurses among the visiting team. This ensured that male clients could be comfortable expressing their health-related issues. The CHNs also requested transport or funding for home visits. The nurses also wanted their home visiting bags equipped with essential tools or equipment to render basic nursing activities during home visits. In addition, the availability of vaccines to visiting staff was enlisted to improve universal coverage.

### Safety measures and protection

The supply of a map to all community workers was strongly advocated for to enable the home visit team to familiarize themselves with the community before their visit. Safety items such as walking boots, umbrellas and raincoats were requested. For effective implementation, nurses suggested a collaboration model with community volunteers, where a trusted community member may be a companion during a home visit. It was also suggested that in the absence of a community companion, nurses must walk in groups as a security measure. The community members also recommended that nurses be given defensive weapons including pepper spray to protect themselves.

“I recommend that they give them a form of security for self-defense. It could be peppering spray, or it could be tasers. There is no way they will be able to carry guns, but if they are given some of these things, they provide for the police, which can be submitted after they are done with the activities for the day. (M1)”

### In-service training

The participating nurses in this study reiterated the need for training and capacity-building in home visiting services. A practice guideline for home visits in the Ghanaian context was also strongly suggested. According to the participants, practice guidelines should outline the scope of practice, and boundaries should be defined. Many also advocated for public education on health-related matters affecting individuals, health professionals and communities.

“For me, the job description needs to be specified better. The communities must be made aware of what we do as visiting nurses. Also, our colleagues and midwives feel that everything that happens must be referred to them as long as it is a pregnant woman. But there are some services we can also give. We are not taking their job. We are just supporting them (G1N5).”

## Discussion

In this study, we explored the challenges of home visits as well as measures to mitigate the challenges in the quest to render effective, efficient and quality health services to individuals, families and communities. Consistent with existing literature [14, 15], this study unearthed challenges encountered by home visit staff and consumers in Ghana. For example, the findings revealed that home visits in Ghana had been bedevilled by several challenges, including transportation, geographical location, access, logistics and finance and sociocultural practices that counter the tenets of home visits. Addressing the challenges, suggestions, including providing in-service training to equip home visit staff to discharge their professional responsibilities and provide safety and security, were made to contribute to effective and efficient home visit services.

Typically, the inadequate resource allocation for home visit purposes exemplifies the general challenges in healthcare delivery in Ghana. Constrained by finance, logistics and human resources from national sources, Ghana’s primary healthcare delivery system has been operating on the support provided by development partners, namely the United States Agency for International Development (USAID), Japan International Cooperation Agency (JICA), World Health Organization and European Union [16]. However, because of restrictions imposed on the application of donor funds [16], it is impossible to make provisions for home visit services when funds are not specifically dedicated to home visit practices.

The challenges relating to transportation and location of communities are arguably common to the Ghanaian context and others across other low- and middle-income countries [17]. Poor road infrastructure and networks are present in several communities across the country. These continuously posed challenges to home visit staff who spend precious time commuting between and within communities. In addition to the discomfort and health problems associated with the poor road network, long travel times often affect the time available for providing healthcare services to clients [1]. Coupled with the above is the location of communities or clients. Unlike in high-income or developed countries, the residential address system is not fully developed, coordinated and easily accessible. This is particularly the case in rural and deprived communities where general infrastructure is limited and poor in supply, including internet services. Google Maps services, which have supported the location of places and households, do not function in rural parts of the country. In a similar study in Kenya, Vedanthan et al. recommend linking Community Health Workers (CHWs) to clients who are hard to reach in rural local communities by mobile phones [18]. This will ensure access to professional care without having to travel to a specific location.

Beyond these challenges is the observation that some clients are motivated to provide a residential address system that is often difficult and sometimes impossible to locate, and by so doing, avoid home visit staff. The motivation could stem from several factors, predominantly sociocultural beliefs that reduce confidence and trust in orthodox healthcare practices but favour traditional healthcare delivery systems and procedures. For example, in some cultural settings in Ghana and other countries, there are traditionally approved intergenerational practices, such as applying hot stones, saliva, ground chalk and towels on the umbilical cord [19, 20]. Because healthcare professionals advise against these practices, there is no incentive to welcome home visit staff who may object to such practices. It is, therefore, unsurprising that lack of trust emerged in this study as another home-visit-avoidance strategy whereby clients deliberately give inaccurate and misleading information about their residence and availability. The findings largely support an earlier observation suggesting that nurses and other home visit staff have difficulties accessing families, households or communities to discuss and advise on topical and sensitive health issues such as contraception and safe sex [17].

The patriarchal orientation of African communities empowers men, who are also the heads of families, as owners and custodians of communities or families [21]. In this framework, their approval is ultimately needed in some instances before life-saving information can be delivered to women and girls by home visit staff. These men can deny home visit access to their communities and homes. Commenting on this development in Uganda, Musoke and colleagues noted several instances of males refusing home visit staff, who have travelled several hours, access to the family or household for health promotion and education activities [17]. The religious and cultural practices and gendered barriers of care often invoke invisible power relations within communities that nurses and other staff who undertake home visits cannot navigate.

Relatedly, the quest for socioeconomic activities has contributed mainly to the unavailability of clients for home visit activities. This worrying observation could be attributed to the economic hardship that forces people to work multiple jobs or longer hours or days. Indeed, poverty is a notable developmental and public health issue in Ghana that affects every aspect of life and forces people to be economically active. Notably, this barrier has also been reported in high-income countries. For instance, reporting on nurses’ experiences in a home visit program in the United States, Beasley Ridings identified unstable lives and limited time resulting from work pressure and inadequate family support as contributing to poor home visit practices [22]. This buttresses the role of socioeconomic dynamics in social determinants of health and utilization of health services. Thus, exposure to education, information and economic income increases the utilization of care services [23].

Addressing the challenges confronting home visit practices, including those discussed above, will contribute to attaining Goal 3 of the United Nation’s SDG and the UHC as envisioned by the WHO. Oleribe et al. suggest context-specific innovative solutions to Africas’ resource deficit healthcare systems [24]. The provision of security and safety should be enhanced. For example, staffing arrangements should be prioritized in addition to prescribing uniforms sensitive to the various communities’ cultural norms. As much as practicable, a home visit team should comprise both males and females to minimize possible sexual harassment of females. A dedicated communication line, accessible to all home visit staff and others such as the Ghana Police Service, should facilitate reporting security and safety issues for prompt action. These and many other recommendations can be achieved through public–private-partnership for the effective implementation and sustainability of home visiting practices [24].

Community engagement, awareness creation and education should be intensified to create awareness of health and wellbeing and the general roles of home visit staff who bridge the formalized healthcare system and communities [25]. To circumvent sociocultural practices occasioned by the patriarchal orientation of Ghanaian communities, males could be made Community Health Ambassadors (CHA). The ambassadorial position should be structured to include training on health issues, home visit staff, the responsibilities of males as custodians and caretakers of communities or households concerning home visits and how they can promote the health of households or families. They should be assigned well-defined tasks, including creating pro-home visit communities through men’s fellowship meetings.

There is evidence that both community or public health nurses and consumer populations require training and awareness creation to increase the knowledge base and competencies about home visits practice [25]. This call could be necessitated by challenges encountered by the home visit staff because of the changing demographics and dynamics of Ghanaian culture and society that could render old practices ineffective and impractical. To support the design of a tailored training program, we propose formative research that explores the needs of home visit staff. These suggestions are in congruence with similar initiatives such as the integrated Community Case Management (iCCM) Health Development Army (HDA) and staff training for the improvement of the Health Extension Program (HEP) Ethiopia [23]. The importance of allocating resources and incentives to the home visit team must be emphasized. In summary, although nurses, midwives and other healthcare professionals can use limited resources to render healthcare services, this is only possible when basic and essential equipment such as thermometers and BP apparatus are available. Thus, efforts should be made to provide home visit staff with operational working tools and safety measures to boost their confidence and morale.

### The implications of the results in the context of home visit guidelines

The Ghanaian health system has benefited from home visit activities organized and delivered by nurses and other health professionals. A dedicated effort to address the existing challenges is pertinent for a home visit practice rooted in an accountability framework and responsive to the health and psychosocial needs of contemporary Ghanaian society to be realized. Based on the findings of the study and the author’s understanding of existing practice arrangements, a dedicated set of home visit guidelines in Ghana that incorporate cultural nuances and provide salient information to ensure effective and efficient service delivery is timely. Home visit guidelines have been developed in high-income countries such as the United States and Canada [22, 26]. Though very useful, it was observed that these guidelines have yet to incorporate sociocultural factors and discussions that are unique to the Ghanaian society and healthcare system. For example, navigating the cultural settings in Ghana requires interaction with layers of community, family and household gatekeepers who yield varying levels of influence on home visit practices.

Similarly, Ghana’s safety and security arrangements, along with transportation and other infrastructural needs for home visit practices, differ substantially from what persists in high-income countries where most guidelines are developed. The efficient addressing system and functioning infrastructural support system for home visit services in the United States are generally lacking in Ghana and other African countries. These observations, coupled with the deficiencies of the existing framework for home visits in Ghana, as noted previously, necessitate the adaptation of guidelines sensitive and responsive to home visit practices in a resource-constrained country such as Ghana. Ethical and human rights frameworks should underpin such guidelines to accommodate the call for a rights-based approach to healthcare service [27]. Based on the study’s findings, home visit guidelines in the Ghanaian context must necessarily streamline service delivery by addressing several salient issues, including service set-up, logistics and human and financial resources.

Alternatively, home visit guidelines that make provisions for the needed resources and tools while tasking the authorities and duty bearers to allocate resources and tools will contribute to effective and efficient home visit practices. If the guidelines also specify roles, duties and responsibilities of the various professional groups working collectively and collaboratively to render home visit services, the avenue for potential or actual role conflict that may occur during the planning, organization and delivery of home visit services may be mitigated. Likewise, home visit guidelines that anticipate and make provisions for safety issues will provide comfort and security to visit staff home. Given the preceding, the home visit guidelines currently being adapted for Ghana will positively change the scope and practice of home visits.

The findings reported in this study may apply to other countries in Africa and other regions such as Asia. This is mainly owing to the similarities in sociocultural practices, economic standing, healthcare practices, infrastructure challenges and addressing systems across low- and middle-income countries [28]. For example, African societies are primarily patriarchal, where men significantly influence household decision-making, including access to and utilization of healthcare services. Based on this and previous studies [17], home visit staff in these settings may need help accessing clients when influence-wielding men are not granted permission. The road infrastructure, network and addressing system could be better in the rural settings of several African countries. As a result, home visit services are essential to residents in rural settings. However, these infrastructural and poor addressing problems posed significant barriers to accessing this vulnerable population to render healthcare services, as reported in this study. Finally, the safety and security arrangement for community health workers, which reflects Ghana’s general security situation, is similar to the security and safety provision instituted for healthcare workers in other African countries [29]. It follows that the proposed home visit guidelines to address the challenges in the Ghanaian context can be extended to countries that share similar characteristics with Ghana.

### Limitations of the study

The study’s findings should be reviewed carefully, considering the following limitations. The lack of representativeness of participants and geographical location could limit the application of the results. The data may be skewed with positive participation bias among the participants. Indeed, we purposively chose the participants for inclusion in the study, raising the possibility that different findings could emerge with other participants. There is also the possibility of recall bias since the participants were not interviewed when experiencing or conducting home visits. Although the study was limited to the Ghanaian context, it is posited that the findings can be extended to other countries that share similar sociocultural and geopolitical systems with Ghana.

## Conclusions

Home visits are a central feature of the Ghanaian healthcare system, contributing significantly to the primary healthcare system. While acknowledging that the findings could not be generalized owing to the study design and issues relating to the representativeness of participants and geographical locations, it is posited that the absence of dedicated home visit guidelines containing pertinent information to support the organization and delivery of home visit activities is a major contributory factor besetting home visit practice. Practice guidelines are essential in healthcare delivery and service evaluation. As noted previously, home visit guidelines specifying the needed resources and addressing safety and security issues will ensure adequate preparation that meets the needs of home visit staff and their clients.

## Data Availability

Not applicable.

## Abbreviations

UHC: Universal healthcare coverage
DPLCAM: Disease prevention life course approach model
CHPS: Community-based health planning strategy
CHOs: Community health officers
RAISE: Research to enhance the adaptation and implementation of health systems guidelines
FGDs: Focus group discussions
CHNs: Community health nurses
CHOs: Community health officers
CHW: Community health worker
CHA: Community health ambassadors
HPM: Health programme managers
RAs: Research assistants
ANC: Antenatal care services
USAID: United states agency for international development
JICA: Japan international cooperation agency
iCCM: Integrated community case management
HAD: Health development army
HEP: Health extension program
BP: Blood pressure
WHO: World health organization
